# Toxicity of Ammonia Stress on the Physiological Homeostasis in the Gills of *Litopenaeus vannamei* under Seawater and Low-Salinity Conditions

**DOI:** 10.3390/biology13040281

**Published:** 2024-04-21

**Authors:** Yuxiu Nan, Meng Xiao, Yafei Duan, Yukai Yang

**Affiliations:** 1 Ocean College, Hebei Agricultural University, Qinhuangdao 066003, China; 2 Key Laboratory of South China Sea Fishery Resources Exploitation & Utilization, Ministry of Agriculture and Rural Affairs, State Key Laboratory of Mariculture Biobreeding and Sustainable Goods, South China Sea Fisheries Research Institute, Chinese Academy of Fishery Sciences, Guangzhou 510300, China; 3 Key Laboratory of Efficient Utilization and Processing of Marine Fishery Resources of Hainan Province, Sanya Tropical Fisheries Research Institute, Sanya 572018, China; 4 Shenzhen Base of South China Sea Fisheries Research Institute, Chinese Academy of Fishery Sciences, Shenzhen 518121, China

**Keywords:** shrimp, gills, ammonia, salinity, physiological function

## Abstract

**Simple Summary:**

Ammonia is a major water quality factor influencing the survival and health of shrimp, among which the gill is the main effector organ for ammonia toxicity. In this study, we explored the toxicity of ammonia stress on the physiological homeostasis in the gills of *Litopenaeus vannamei* under seawater and low-salinity conditions. This study included four groups, namely the SC group (ammonia-N 0 mg/L, salinity 30‰), SAN group (ammonia-N 10 mg/L, salinity 30‰), LC group (ammonia-N 0 mg/L, salinity 3‰), and LAN group (ammonia-N 10 mg/L, salinity 3‰). The ammonia stress lasted for 14 days. The results show that ammonia stress caused the severe contraction of gill filaments and the deformation or even rupture of gill vessels. Ammonia stress could also influence the redox, ER function, apoptosis, detoxification, energy metabolism, and osmoregulation of the shrimp gills. These results are helpful to analyze the toxicological mechanism of ammonia stress on the seawater- and low salinity-cultured shrimp.

**Abstract:**

Ammonia is a major water quality factor influencing the survival and health of shrimp, among which the gill is the main effector organ for ammonia toxicity. In this study, we chose two types of *Litopenaeus vannamei* that were cultured in 30‰ seawater and domesticated in 3‰ low salinity, respectively, and then separately subjected to ammonia stress for 14 days under seawater and low-salinity conditions, of which the 3‰ low salinity-cultured shrimp were domesticated from the shrimp cultured in 30‰ seawater after 27 days of gradual salinity desalination. In detail, this study included four groups, namely the SC group (ammonia-N 0 mg/L, salinity 30‰), SAN group (ammonia-N 10 mg/L, salinity 30‰), LC group (ammonia-N 0 mg/L, salinity 3‰), and LAN group (ammonia-N 10 mg/L, salinity 3‰). The ammonia stress lasted for 14 days, and then the changes in the morphological structure and physiological function of the gills were explored. The results show that ammonia stress caused the severe contraction of gill filaments and the deformation or even rupture of gill vessels. Biochemical indicators of oxidative stress, including LPO and MDA contents, as well as T-AOC and GST activities, were increased in the SAN and LAN groups, while the activities of CAT and POD and the mRNA expression levels of antioxidant-related genes (*nrf2*, *cat*, *gpx*, *hsp70*, and *trx*) were decreased. In addition, the mRNA expression levels of the genes involved in ER stress (*ire1* and *xbp1*), apoptosis (*casp-3*, *casp-9*, and *jnk*), detoxification (*gst*, *ugt*, and *sult*), glucose metabolism (*pdh*, *hk*, *pk*, and *ldh*), and the tricarboxylic acid cycle (*mdh*, *cs*, *idh*, and *odh*) were decreased in the SAN and LAN groups; the levels of electron-transport chain-related genes (*ndh*, *cco*, and *coi*), and the *bip* and *sdh* genes were decreased in the SAN group but increased in the LAN group; and the level of the *ATPase* gene was decreased but the *cytc* gene was increased in the SAN and LAN groups. The mRNA expression levels of osmotic regulation-related genes (*nka-β*, *ca*, *aqp* and *clc*) were decreased in the SAN group, while the level of the *ca* gene was increased in the LAN group; the *nka-α* gene was decreased in both two groups. The results demonstrate that ammonia stress could influence the physiological homeostasis of the shrimp gills, possibly by damaging the tissue morphology, and affecting the redox, ER function, apoptosis, detoxification, energy metabolism, and osmoregulation.

## 1. Introduction

The Pacific white shrimp *Litopenaeus vannamei* is a global economic shrimp, which exhibits a wide range of salinity tolerance. *L. vannamei* can survive in salinity levels ranging from 0.5‰ to 40‰, making it suitable for cultivation in low-salinity areas. As a result, there has been a significant expansion of shrimp farming [1]. Shrimp culture has been plagued by diseases for a long time, and environmental stress is an important cause of disease occurrence [2]. Ammonia is a frequent environmental impact factor in farming water [3], which is mainly the result of the decomposition of residues and feces, as well as the metabolites of aquatic animal proteins and amino acids [4]. Ammonia in water usually occurs as ammonium salt (NH_4_^+^) and free ammonia (NH_3_), which can be converted into each other [5]. Among them, NH_3_ is fat-soluble and easily penetrates the cell membrane, which can directly damage the gill epidermal cells of shrimp, and reduce the oxygen-carrying ability of hemocyanin [6,7]. Due to high-density aquaculture, high concentrations of ammonia in aquaculture water occur frequently, which seriously affects the growth, development, immunity, and disease resistance of shrimp [8].

Shrimp mainly depend on its non-specific immunity to resist environmental stress [9]. It was reported that ammonia stress decreased superoxide dismutase (SOD) activity in the hemolymph and hepatopancreas of *L. vannamei*, consequently leading to a notable increase in malondialdehyde (MDA) content in the hepatopancreas [10,11]. Long et al. [12] observed marked alterations in the total antioxidant capacity (T-AOC), antioxidant enzyme activity, and mRNA expression levels of the heat shock protein 70 (*hsp70*) and thioredoxin (*trx*) genes in *L. vannamei* when subjected to elevated levels of both ammonia and salinity stress. Ou et al. [13] found that acute exposure to ammonia increased the mRNA expression level of the kelch-like ECH-associated protein 1 (*keap1*) gene in the hepatopancreas of *Marsupenaeus japonicus*, whereas the expression of the nuclear factor erythroid 2-related factor 2 (*nrf2*) gene was down-regulated. Liang et al. [11] showed that ammonia stress significantly increased the mRNA levels of endoplasmic reticulum (ER) stress markers, including the immunoglobulin heavy chain binding protein (*bip*), transcription factor 4 (*atf4*), and x-box binding protein 1 (*xbp1*) in the hepatopancreas of *L. vannamei*. Additionally, ammonia exposure also promoted the apoptosis of hepatopancreas and hemocytes in *L. vannamei*, and induced mRNA expression changes in the apoptosis-related gene caspase 3 (*casp-3*) in the hepatopancreas of *M. japonicus* [7,11,13].

Ammonia stress can affect the metabolic function of shrimp. For example, after *L. vannamei* were exposed to 1.61 mg/L NH_3_ stress, the content of lactic acid (LA) in the hemolymph was increased initially before subsequently diminishing and the activities of the hexokinase (HK) and succinate dehydrogenase (SDH) in the hepatopancreas were decreased, while the activities of the phosphofructokinase (PFK) and pyruvate kinase (PK) were increased, indicating that ammonia stress could disrupt the metabolic function of *L. vannamei* [14]. Under acute ammonia stress, the glycolytic enzymes’ activity and glucose and lactic acid contents were increased in *L. vannamei*, indicating that the anaerobic metabolism of shrimp was enhanced in response to stress [15]. Furthermore, ammonia stress can influence the osmotic regulation ability of shrimp. For example, Chen and Nan [16] observed that the activities of the total ATP synthase (ATPase) and Na^+^/K^+^-ATPase in the gills of *Penaeus chinensis* were increased under 5.043 mg/L ammonia-N stress, but were decreased under 10.106 and 20.093 mg/L ammonia-N stress.

Gills are osmotic adjustment organs for ion exchange and acid–base balance maintenance in shrimp [17]. Shrimp is an ammonia-excreting organism, and more than 60% of the nitrogen-containing excreta in the body are excreted from the gills in the form of ammonia nitrogen. Due to the direct contact between the gill tissue and the water environment, when the concentration of ammonia in the water is too high, the gill tissue will be affected first [18]. However, the influence mechanism of ammonia exposure on the physiological function in the gills of shrimp cultured in seawater and low salinity is still unknown.

Therefore, in this study, we chose two types of *L. vannamei* that were cultured in 30‰ seawater and domesticated in 3‰ low salinity, respectively, and then separately subjected to ammonia stress for 14 days under seawater and low-salinity conditions. Finally, the physiological response characteristics of the *L. vannamei* gills under ammonia stress were explored at multiple levels, including the tissue structure, oxidative stress, ER stress, apoptosis, detoxification, energy metabolism, osmotic adjustment, etc. Our results can provide theoretical insights on the ammonia toxicity of shrimp under seawater and low-salinity conditions.

## 2. Materials and Methods

### 2.1. Shrimp Materials

In this study, two types of *L. vannamei* that were cultured in 30‰ seawater and domesticated in 3‰ low salinity, separately, weighing on averaging 9.9 ± 0.2 g, were randomly selected for the ammonia stress experiment. The 3‰ low salinity-cultured shrimp were domesticated from the shrimp cultured in 30‰ seawater after 27 days of gradual salinity desalination. The healthy shrimp were temporarily cultured in tanks for 7 days. During the temporary culture period, the temperature of the culture water was 26 ± 0.5 °C, the pH was 7.9–8.1, and continuous aeration was performed for 24 h. Half of the water was renewed daily, compound feed was used to feed shrimp according to 5% of its body weight, and the residues and feces were removed punctually.

**Figure 1:**
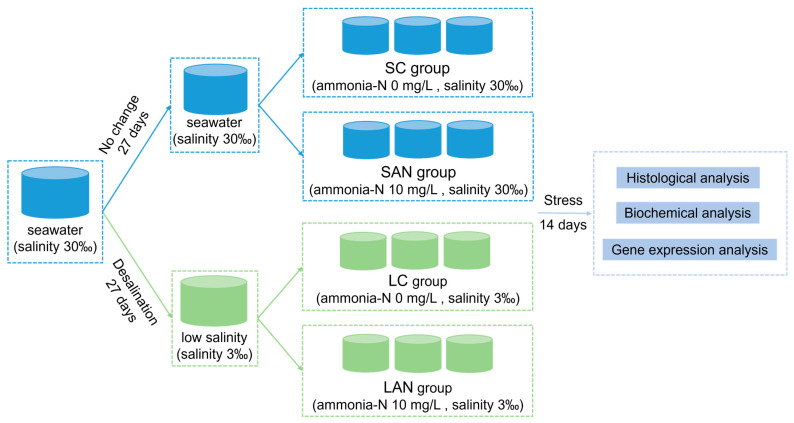
Schematic representation of the experimental design in this study.

The experimental design is shown in Figure 1. In detail, these two types of the seawater- and low salinity-cultured shrimp were from the same parent, and were cultured in the same indoor pond since the postlarvae 10 stage. Before the stress experiment, the 30‰ seawater-cultured shrimp was divided into two ponds, one of which was always maintained at 30‰ seawater, while the other pond was gradually desalinated to 3‰ salinity. The salinity desalination method was as follows: the initial salinity was 30‰, and the salinity was reduced by 1‰ with aerated tap water every day. After 27 days of desalination, the water salinity reached 3‰ and the desalination was completed. All of the shrimp were temporarily cultured in a tank for 7 days, and then exposed to ammonia for 14 days, and the gill samples were collected for analysis.

### 2.2. Ammonia Stress Experiment and Sampling

Following one week of temporary culture in 30‰ seawater and 3‰ low-salinity, separately, the shrimp were divided into four groups at random: the seawater control group (SC), seawater ammonia-exposure group (SAN), low-salinity control group (LC), and low-salinity ammonia-exposure group (LAN). There were 3 replicate tanks in each group, and each tank had 30 shrimp. The rearing water of the SC and SAN groups was normal seawater with 30‰ salinity, while that of the LC and LAN groups was 3‰ low-salinity water achieved by adding fresh water to seawater. According to the related research on the ammonia stress of *L. vannamei* [10,19], the ammonia-N concentration in the SAN and LAN groups was set at 10 mg/L, which was adjusted by directly adding NH_4_Cl into the water. There were four groups, namely the SC group (ammonia-N 0 mg/L, salinity 30‰), SAN group (ammonia-N 10 mg/L, salinity 30‰), LC group (ammonia-N 0 mg/L, salinity 3‰), and LAN group (ammonia-N 10 mg/L, salinity 3‰). Half of the water in each container was exchanged daily, and the ammonia-N concentration was determined and adjusted every 4–6 h. Except for differences in the salinity or ammonia-N concentration, the daily management of the shrimp in each group during the experiment was consistent with that during the temporary culture.

Samples were collected after two weeks of ammonia exposure. Five shrimp gills were collected from each tank and stored at −80 °C for the detection of biochemical indexes. The gills of three shrimp were collected from each tank, put in RNA-preserving solution (RNAFollow, NCM Biotech, Suzhou, China) at 4 °C for a whole day, and then preserved at −80 °C for the analysis of genes’ expression. For subsequent histological analysis, the gills of three shrimp in each tank were fixed in 4% paraformaldehyde.

### 2.3. Histological Analysis

After being fixed for 24 h in 4% paraformaldehyde reagente (Biosharp, Guangzhou, China), the gill tissue was taken out and rinsed under flowing water in order to remove the residual 4% paraformaldehyde, and gradually dewatered using 70%, 80%, 90%, and 100% ethyl alcohol by volume. It was then placed in xylene to make it transparent, and the tissue was then embedded in melted paraffin wax to harden the tissue blocks and facilitate slicing. The embedded tissue blocks were cut into 4 μm tissue sections and stained with hematoxylin and eosin. Lastly, the tissue was dried at ambient temperature and sealed with neutral resin for long-term preservation. The morphological changes in the tissue were observed under a microscope (NIKON Eclipse ci, Nikon, Tokyo, Japan) and photographed (NIS_F_Ver43000_64bit_E, Nikon, Tokyo, Japan).

### 2.4. Biochemical Analysis

The preserved gill tissue samples were thawed on ice, and then rinsed with pre-cooled 0.9% saline solution to remove the tissue fluid. After drying with filter paper, the gill tissue mass was accurately weighed, and 9 times the volume of 0.9% saline solution (Biosharp, Guangzhou, China) was added. Tissue homogenizer (TissueLyser II, Germany Qiagen, Berlin, Germany) was used to prepare 10% tissue homogenate at 4 °C. After centrifugation at 3500 r/min for 10 min, a homogenate supernatant was obtained for the determination of biochemical indicators. Total protein (TP), catalase (CAT), glutathione peroxidase (GPx), glutathione S-transferase (GST), peroxidase (POD), lipid peroxide (LPO), MDA, and T-AOC were detected by the same batch kits (Jiancheng, Ltd., Nanjing, China), and were analyzed using a microplate reader instrument (Infinite M200 Pro, Switzerland TECAN, Zurich, Switzerland). The specific operation methods refer to the specifications.

### 2.5. Gene Expression Analysis

RNA was isolated from the gill tissues of the shrimp using the Trizol reagent (Invitrogen, Beijing, China). RNA quality was assessed using 1.0% agarose gel electrophoresis, and the Nanodrop 2000 was used to determine the RNA concentration. Reverse transcription was performed to convert the RNA into cDNA using the Servicebio^®^ RT First Strand cDNA Synthesis Kit (Servicebio, Wuhan, China), and kept for subsequent use at −80 °C. The cDNA nucleotide sequences of the target genes and the internal reference gene of *L. vannamei* were downloaded from the NCBI database. The primer sequences were designed using Primer Premier 5.0 (Table S1), and the forward and reverse specific primers were synthesized by Shanghai Sangon Biotech Co., Ltd. (Shanghai, China). The amplification efficiency of the primers was verified using the standard curve. The internal reference gene of *L. vannamei* was the *β-actin* gene. The SYBR Green Pro Taq HS Premix kit (Accurate Biotechnology Co., Ltd., Hunan, China) was adopted for qPCR operation in a real-time fluorescence quantitative PCR testing apparatus (Likang CG-02, Shanghai, China). The quantitative real-time PCR reaction mixture amount was 15 μL, comprising 7.5 μL SYBR Green Pro Taq HS Premix (2×), 5.3 μL RNase-free water, 0.6 μL 10 μmol/L forward primer, 0.6 μL 10 μmol/L reverse primer, and 1.0 μL cDNA. The qPCR reaction program was 95 °C for 30 s, 40 cycles of 95 °C for 5 s, and 60 °C for 30 s. The relative mRNA expression levels of the genes were calculated following the method of Livak and Schmittgen [20]. The gene expression levels of the SAN, LC, and LAN groups were all expressed as the fold-change in the SC group, referring to the SC group as the control.

### 2.6. Statistical Analysis

All the data were presented as mean ± standard error (SE), one-way analysis of variance (ANOVA) was conducted using SPSS 27.0, and a post-hoc test was performed using the least significant difference (LSD) and Duncan methods, with *p* < 0.05 indicating a significant difference.

## 3. Results

### 3.1. The Histological Morphology Changes in the Shrimp Gills

**Figure 2:**
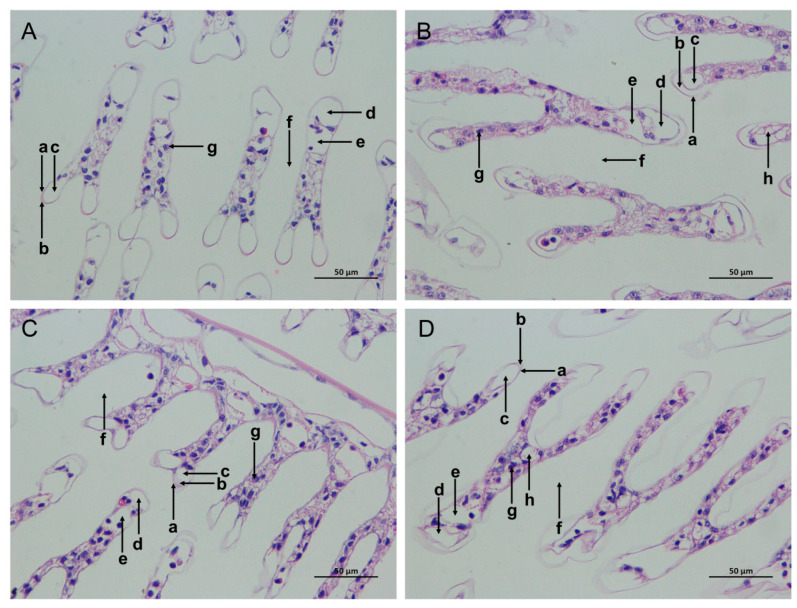
Effects of ammonia stress on the gill tissue morphology of *L. vannamei* under seawater and low-salinity conditions. (**A**) The SC group; (**B**) the SAN group; (**C**) the LC group; (**D**) the LAN group. a: cuticle; b: epithelial cells; c: subcutaneous space; d: afferent vessel; e: efferent vessel; f: diaphragm; g: blood cells; h: vacuoles; 400× magnification.

The gill tissue boundaries of the SC and LC groups were clear, and the morphological structure of the epithelial cells and stratum corneum was normal (Figure 2A,C). In comparison with the SC group, the SAN group had blurred gill tissue boundaries, damaged cuticles, decreased blood cells, widened subcutaneous spaces, and increased cavity cavitations (Figure 2B). In comparison with the LC group, the gill tissue of the LAN group resembled that of the SAN group, with a serious contraction of the gill filaments, and the deformation and even rupture of the gill vessels (Figure 2D).

### 3.2. Changes in Oxidative Stress Biochemical Parameters in the Shrimp Gills

**Figure 3:**
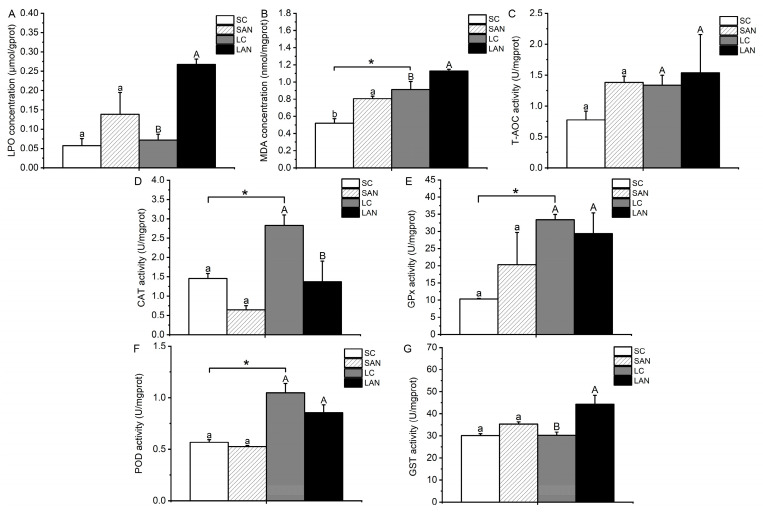
Effects of ammonia stress on the biochemical indexes of oxidative stress in the gills of *L. vannamei* under seawater and low-salinity conditions. (**A**) LPO content; (**B**) MDA content; (**C**) T-AOC activity; (**D**) CAT activity; (**E**) GPx activity; (**F**) POD activity; (**G**) GST activity. Different lowercase letters show significant differences (*p* < 0.05) between the two groups under 30‰ salinity. Different capital letters show significant differences (*p* < 0.05) between the two groups under 3‰ salinity. * represents significant differences (*p* < 0.05) between the SC and LC groups.

In comparison with the SC group, the contents of LPO and MDA and the activities of GPx, GST, and T-AOC were increased in the SAN group, whereas the activities of CAT and POD were decreased. The only variable that differed significantly was the MDA content (*p* < 0.05). In comparison with the LC group, the contents of LPO and MDA and the activities of GST and T-AOC were increased in the LAN group, and only the T-AOC activity was not statistically different (*p* > 0.05); the activities of CAT, GPx, and POD were decreased in the LAN group, but only the CAT activity was statistically different (*p* < 0.05). Furthermore, it was observed that the LC group had significantly greater activities of CAT, GPx, POD, and MDA content in comparison to the SC group (*p* < 0.05) (Figure 3).

### 3.3. Changes in Antioxidant-Relevant Gene Expression in the Shrimp Gills

**Figure 4:**
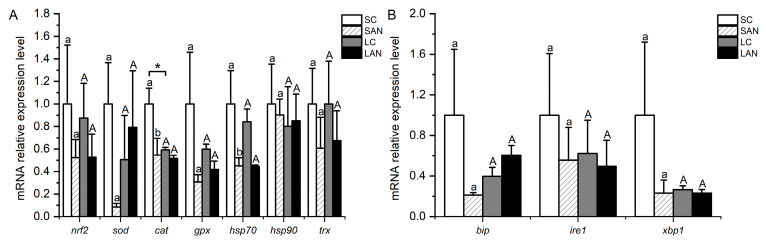
Effects of ammonia stress on the antioxidant and endoplasmic reticulum stress-related genes’ expression in the gills of *L. vannamei* under seawater and low-salinity conditions. (**A**) Oxidative stress-related genes; (**B**) ER stress-related genes. Different lowercase letters show significant differences (*p* < 0.05) between the two groups under 30‰ salinity. Different capital letters show significant differences (*p* < 0.05) between the two groups under 3‰ salinity. * represents significant differences (*p* < 0.05) between the SC and LC groups.

In comparison with the SC group, the relative mRNA expression levels of antioxidant-relevant genes, including *nrf2*, *sod*, *cat*, *gpx*, *hsp70*, heat shock protein 90 (*hsp90*), and *trx*, were down-regulated in the SAN group, but only the level of the *cat* and *hsp70* genes was statistically different (*p* < 0.05). In comparison with the LC group, the mRNA expression levels of the *nrf2*, *cat*, *gpx*, *hsp70*, and *trx* genes were down-regulated in the LAN group, while there was an up-regulation in the levels of the *sod* and *hsp90* genes with no significant difference (*p* > 0.05). Furthermore, the mRNA expression level of the *cat* gene was significantly lower in the LC group when compared with the SC group (Figure 4A).

### 3.4. Changes in ER Stress-Relevant Genes’ Expression in the Shrimp Gills

In comparison with the SC group, the relative mRNA expression levels of ER stress-relevant genes, including *bip*, inositol-requiring enzyme 1 (*ire1*), and *xbp1*, were down-regulated in the SAN group, although the differences were not significant (*p* > 0.05). In comparison with the LC group, the mRNA expression levels of the *ire1* and *xbp1* genes were down-regulated in the LAN group, while the *bip* gene was up-regulated; nevertheless, there was no significant difference (*p* > 0.05) (Figure 4B).

### 3.5. Changes in Apoptosis-Relevant Genes’ Expression in the Shrimp Gills

**Figure 5:**
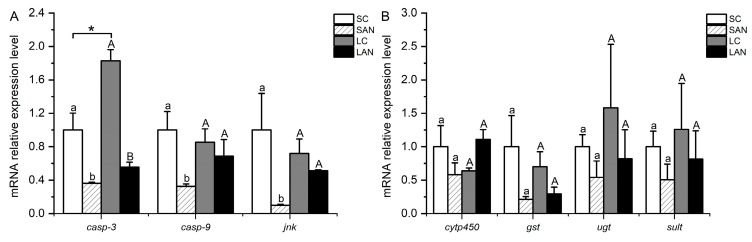
Effects of ammonia stress on the apoptosis and detoxification metabolism-related genes’ expression in the gills of *L. vannamei* under seawater and low-salinity conditions. (**A**) Apoptosis-related genes; (**B**) detoxification metabolism-related genes. Different lowercase letters show significant differences (*p* < 0.05) between the two groups under 30‰ salinity. Different capital letters show significant differences (*p* < 0.05) between the two groups under 3‰ salinity. * represents significant differences (*p* < 0.05) between the SC and LC groups.

In comparison with the SC group, the relative mRNA expression levels of apoptosis-relevant genes, including *casp-3*, caspase-9 (*casp-9*), and c-jun n-terminal kinase (*jnk*), were significantly down-regulated in the SAN group (*p* < 0.05). In comparison with the LC group, the mRNA expression levels of the *casp-3*, *casp-9*, and *jnk* genes were down-regulated in the LAN group, but only the level of the *casp-3* gene was significantly different (*p* < 0.05). Furthermore, it was observed that the LC group had a significantly higher expression level of the *casp-3* gene than the SC group (*p* < 0.05) (Figure 5A).

### 3.6. Changes in Detoxification-Relevant Genes’ Expression in the Shrimp Gills

In comparison with the SC group, the relative mRNA expression levels of detoxification-related genes, including cytochrome p450 (*cytp450*), *gst*, udp-glucuronosyltransferase (*ugt*), and sulfotransferase (*sult*), were down-regulated in the SAN group, but no significant difference was found (*p* > 0.05). In comparison with the LC group, the mRNA expression levels of the *gst*, *ugt*, and *sult* genes were down-regulated in the LAN group, whereas the expression of the *cytp450* gene was up-regulated; however, there was no significant difference (*p* > 0.05) (Figure 5B).

### 3.7. Changes in Energy Metabolism-Related Genes’ Expression in the Shrimp Gills

**Figure 6:**
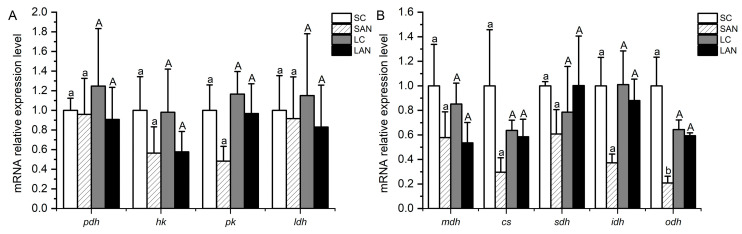
Effects of ammonia stress on the energy metabolism-related genes’ expression in the gills of *L. vannamei* under seawater and low-salinity conditions. (**A**) Glucose metabolism-related genes; (**B**) tricarboxylic acid cycle-related genes. Different lowercase letters show significant differences (*p* < 0.05) between the two groups under 30‰ salinity. Different capital letters show significant differences (*p* < 0.05) between the two groups under 3‰ salinity.

In terms of glucose metabolism-related genes, in comparison with the SC group, the mRNA expression levels of the *hk* and *pk* genes were down-regulated in the SAN group, but no significant difference was found (*p* > 0.05). In comparison with the LC group, the mRNA expression levels of the pyruvate dehydrogenase (*pdh*), *hk*, *pk*, and lactate dehydrogenase (*ldh*) genes were down-regulated in the LAN group; nevertheless, no significant difference was found (*p* > 0.05) (Figure 6A).

In terms of tricarboxylic acid cycle-related genes, in comparison with the SC group, the mRNA expression levels of the malate dehydrogenase (*mdh*), citrate synthase (*cs*), *sdh*, isocitrate dehydrogenase (*idh*), and 2-oxoglutarate dehydrogenase (*odh*) genes were down-regulated in the SAN group, but only the level of the *odh* gene had significant differences (*p* < 0.05). In comparison with the LC group, the mRNA expression levels of the *mdh* and *idh* genes were down-regulated in the LAN group, while there was an up-regulation in the level of the *sdh* gene; nevertheless, no significant difference was found (*p* > 0.05) (Figure 6B).

**Figure 7:**
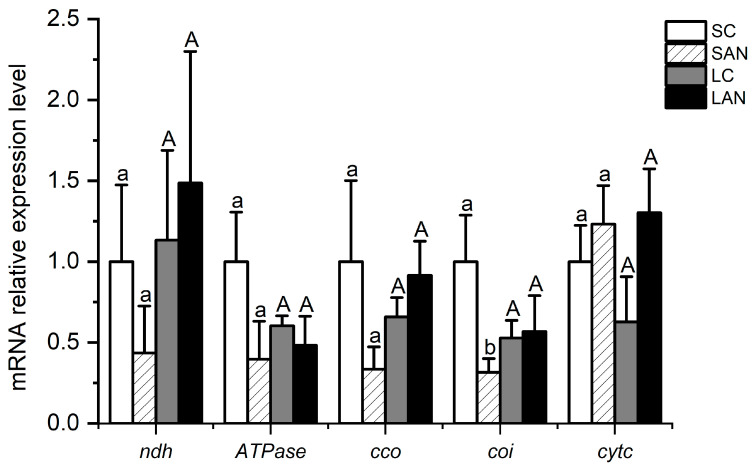
Effects of ammonia stress on the electron transport chain-related genes’ expression in the gills of *L. vannamei* under seawater and low-salinity conditions. Different lowercase letters show significant differences (*p* < 0.05) between the two groups under 30‰ salinity. Different capital letters show significant differences (*p* < 0.05) between the two groups under 3‰ salinity.

In terms of electron transport chain-related genes, in comparison with the SC group, the mRNA expression levels of the NADH dehydrogenase (*ndh*), *ATPase*, cytochrome c oxidase (*cco*), and cytochrome oxidase I (*coi*) genes were down-regulated in the SAN group, whereas the level of the cytochrome c (*cytc*) gene was up-regulated, but only the level of the *coi* gene had significant differences (*p* < 0.05). In comparison with the LC group, the mRNA expression levels of the *ndh*, *cco*, and *cytc* genes were up-regulated in the LAN group, whereas there was a decrease in the level of the *ATPase* gene; nevertheless, no significant difference was found (*p* > 0.05) (Figure 7).

### 3.8. Changes in Osmoregulation-Relevant Genes’ Expression in the Shrimp Gills

**Figure 8:**
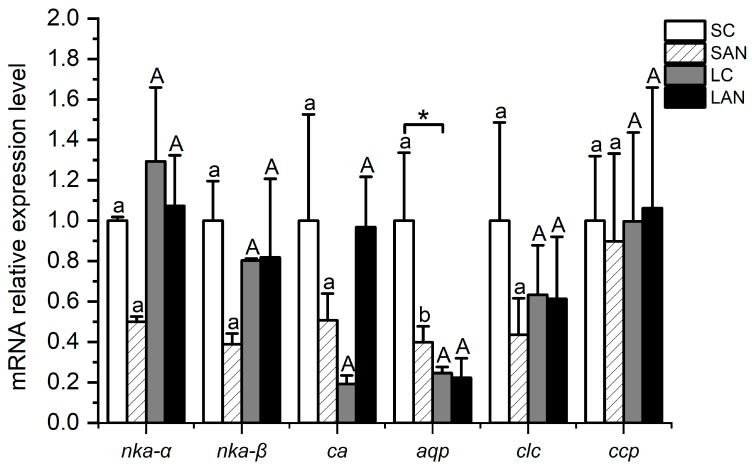
Effects of ammonia stress on the osmotic regulation-related genes’ expression in the gills of *L. vannamei* under seawater and low-salinity conditions. Different lowercase letters show significant differences (*p* < 0.05) between the two groups under 30‰ salinity. Different capital letters show significant differences (*p* < 0.05) between the two groups under 3‰ salinity. * represents significant differences (*p* < 0.05) between the SC and LC groups.

In comparison with the SC group, the relative mRNA expression levels of osmotic regulation-relevant genes, including the Na+/K+-ATPase α subunit (*nka-α*), Na+/K+-ATPase β subunit (*nka-β*), carbonic anhydrase (*ca*), aquaporin (*aqp*), chloride channel protein 2 (*clc*), and calcium channel protein 1 (*ccp*), were down-regulated in the SAN group, but only the level of the *aqp* gene had significant differences (*p* < 0.05). In comparison with the LC group, the level of the *nka-α* gene was down-regulated in the LAN group, while the level of the *ca* gene was up-regulated; nevertheless, there was no significant difference (*p* > 0.05). In addition, the mRNA expression level of the *aqp* gene in the LC group was significantly lower than that of the SC group (*p* < 0.05) (Figure 8).

## 4. Discussion

Ammonia is a common influencing factor in shrimp culture environments [3]. An excessive concentration of ammonia in the water will influence the growth, development, immunity, and disease resistance of shrimp, but the mechanism of the impact of ammonia stress on the physiological function in the gills of the seawater- and low salinity-cultured shrimp is not clear. In this study, we found that ammonia exposure could influence the morphological structure of the gills of *L. vannamei* under seawater and low-salinity conditions, which might have an effect on their physiological homeostasis. Hence, we comprehensively evaluated the physiological responses in the gills of *L. vannamei* to ammonia stress from the points of view of oxidative stress, ER stress, apoptosis, detoxification, energy metabolism, and osmotic regulation.

Oxidative stress is one of the toxic influences of environmental contaminants on shrimp [21]. LPO and MDA contents can reflect the degree of oxidative stress injury to the organism [22]. In this study, LPO and MDA contents were induced in the gills of the seawater- and low salinity-cultured shrimp, showing that oxidative stress happened in the gills following 14 days of ammonia stress. The measurement of the antioxidant defense system’s capability requires the assessment of T-AOC, which serves as an essential indicator [23]. Some key antioxidant enzymes, for instance SOD, CAT, GPx, POD, and GST, are important for defending the organism against oxidative stress [24,25]. Nrf2 can bind with antioxidant-response element (ARE) to adjust the expression of antioxidant enzyme genes [26]. In this study, after 14 days of ammonia stress, the activities of T-AOC and GST were increased in the gills of the seawater- and low salinity-cultured shrimp, while CAT and POD activities and the mRNA expression levels of the *nrf2*, *cat*, and *gpx* genes were decreased. The activity of GPx was increased in the seawater-cultured shrimp but decreased in the low salinity-cultured shrimp, while the expression of the *sod* gene was opposite to the change in GPx activity. These phenomena indicate that ammonia exposure led to oxidative stress injury in the gills of the seawater- and low salinity-cultured shrimp, resulting in the dysfunction of the antioxidant enzyme system and Nrf2 signaling, and different antioxidant enzymes had different response characteristics to ammonia stress.

HSPs are important stress proteins in organism, which can exert antioxidant functions [27]. Trx is a redox protein that can be used as the zymolyte of an antioxidant protein to participate in cellular redox processes [28]. In this study, the mRNA expression levels of the *hsp70* and *trx* genes were decreased in the gills of the seawater- and low salinity-cultured shrimp after 14 days of ammonia stress. The expression of the *hsp90* gene was decreased in the seawater-cultured shrimp, but was slightly increased in the low salinity-cultured shrimp. These phenomena show that ammonia stress had negative effects on the stress proteins in the gills of the seawater- and low salinity-cultured shrimp, which would weaken the anti-stress ability of the organism.

When the organism is stimulated by the external environment, it will induce ER stress, thus starting the UPR to keep ER homeostasis and restore cell function [29]. When ERS occurs, the protein kinase IRE1 dissociates and activates with the molecular chaperone Bip, splicing the coding transcription factor XBP1 to form XBP1s, and then corrects the ER homeostasis by inducing the gene expression of ER folding and the related protein degradation [30]. In this study, following two weeks of ammonia stress, the expressions of the *ire1* and *xbp1* genes were declined in the gills of the seawater- and low salinity-cultured shrimp. The level of the *bip* gene was declined in the seawater-cultured shrimp but increased in the low salinity-cultured shrimp. It is speculated that ammonia stress attracted the normal ER stress process in the gills of the seawater- and low salinity-cultured shrimp, and interfered with the regulation process of ER functional homeostasis.

Apoptosis is a programmed cell death controlled by genes [31]. When cells are stimulated by the environment, the apoptosis complex initiates the apoptosis cascade reaction and activates Casp-9, and then cuts the downstream Casp-3 to achieve apoptosis [32]. JNK can mediate apoptosis by regulating key apoptotic factors [33]. In this study, following two weeks of ammonia stress, the expression levels of the *casp-3*, *casp-9*, and *jnk* genes were decreased in the gills of the seawater- and low salinity-cultured shrimp, indicating that ammonia stress inhibited the apoptosis function of the gills, which was adverse to the maintenance of the homeostasis in the organism.

In response to environmental stress, aquatic animals can mitigate bodily damage by activating their detoxification metabolic system. Organisms can use phase I detoxification enzymes (cytP450, etc.) and phase II detoxification enzymes (GST, UGT, SULT, etc.) to convert harmful substances in cells into water-soluble metabolites in turn, and finally discharge them out of the cell [34]. In this study, following two weeks of ammonia stress, the expressions of the *gst*, *ugt*, and *sult* genes were decreased in the gills of the seawater- and low salinity-cultured shrimp. The expression level of the *cytp450* gene was decreased in the seawater-cultured shrimp but increased in the low salinity-cultured shrimp. This phenomenon shows that ammonia stress reduced the detoxification capacity of the shrimp gills under seawater- and low salinity-cultured conditions. Although the expression of the *cytp450* gene in the gills of low salinity-cultured shrimp was up-regulated, the down-regulation of the phase II metabolic enzyme expression was also not conducive to the detoxification metabolism ability of the gill tissues.

Energy metabolism is the process of releasing energy after nutrients in the organism are catalyzed by various enzymes. Glucose metabolism is one of the pathways of energy metabolism in aquatic animals, among which glycolysis is an important link of glucose metabolism that decomposes glucose or glycogen to keep the energy provision of the body [35,36]. PDH, HK, and PK are key rate-limiting enzymes in glycolysis, and LDH is an important coenzyme in glucose metabolism [14,37]. In this study, following two weeks of ammonia stress, the expressions of the *hk*, *pk*, and *ldh* genes were decreased in the gills of the seawater- and low salinity-cultured shrimp, and the level of the *pdh* gene only was decreased in the low-salinity shrimp, indicating that ammonia stress reduced the glucose metabolism in the gills of the shrimp. The tricarboxylic acid cycle is a chemical reaction catalyzed by a series of enzymes such as MDH, CS, SDH, IDH, and ODH, which generates energy by oxidizing acetyl-CoA formed from the decomposition of lipids or other nutrients [38]. In this study, following two weeks of ammonia stress, the expression levels of the *mdh*, *cs*, *idh,* and *odh* genes were decreased in the gills of the seawater- and low salinity-cultured shrimp. The level of the *sdh* gene was decreased in the seawater-cultured shrimp but increased in the low salinity-cultured shrimp. These changes show that ammonia stress might interfere with the normal function of the shrimp gill tissue by reducing the gene expression of tricarboxylic acid cycle-related enzymes.

The electron transport chain, also known as the respiratory chain, is a successive reaction system composed of a sequence of hydrogen and electron transfer reactions organized in a certain order in mitochondria [39]. NDH and CCO are the first enzyme and the terminal enzyme in the respiratory chain, respectively [40]. ATPase can catalyze the synthesis of ATP in the process of energy metabolism [41]. COI and CytC are important proteins and electron carriers in the electron transport chain, respectively [42]. In this study, following two weeks of ammonia stress, the expression of the *ATPase* gene was decreased in gills of the seawater- and low salinity-cultured shrimp, while the level of the *cytc* gene was enhanced. The levels of the *ndh*, *cco*, and *coi* genes were decreased in marine shrimp but increased in low-salinity shrimp. This phenomenon shows that ammonia stress influenced the balance of energy generation in mitochondria by disrupting the gene expression of electron transport chain in the shrimp gills, while the respiratory chain of the shrimp would have different response characteristics to ammonia stress. In summary, ammonia stress could interfere with the energy metabolism homeostasis in the gills of the seawater- and low salinity-cultured shrimp by affecting the gene expression of key nodes in the glucose metabolism, tricarboxylic acid cycle, and electron transport chain.

Osmotic regulation is a regulatory mechanism that occurs when the homeostasis of aquatic animals is unbalanced. NKA is a key enzyme in ion transport during osmotic regulation [43]. It is ordinarily in charge of the positive membrane transport of Na^+^ and K^+^ on both sides of the cell [44]. CA can regulate the ion concentration and acid–base balance [45]. In this study, following two weeks of ammonia stress, the expression of the *nka-α* gene was decreased in the gills of the seawater- and low salinity-cultured shrimp. However, the level of the *nka-β* gene only was decreased in the seawater-cultured shrimp. The level of the *ca* gene was declined in the seawater-cultured shrimp, but increased in the low salinity-cultured shrimp. It is inferred that ammonia stress interfered with the osmoregulation function of the organisms by affecting the expression of osmotic regulation-related enzymes and genes in the shrimp gills.

AQP is an intrinsic membrane protein capable of transporting small molecular solutes such as water, glycerol, and urea [46,47]. CLC and CCP principally participate in the membrane transport of Cl^−^ and Ca^+^, respectively [21]. In this study, following two weeks of ammonia stress, the expressions of the *aqp* and *clc* genes were substantially decreased in the gills of the seawater-cultured shrimp, while the changes were not significant in the low salinity-cultured shrimp. The expression of the *ccp* gene had no significant changes in the seawater- and low salinity-cultured shrimp. This phenomenon indicates that ammonia stress could interfere with the osmoregulation function of the organisms by reducing the gene expression of water and chloride channel proteins in the gills of the seawater- and low salinity-cultured shrimp.

## 5. Conclusions

This study revealed that ammonia stress influenced the morphological structure of the gills of the seawater- and low salinity-cultured shrimp, and affected the redox and ER functional homeostasis by inducing stress. Furthermore, ammonia stress also reduced the apoptosis, detoxification metabolism, energy metabolism, and osmotic regulation function in the gills. By contrast, the gills of the low salinity-cultured shrimp had the stronger stress responses to ammonia exposure. Although the changes in some indexes in this study were not significant, the results suggest that the harm of ammonia to the gills of the seawater- and low salinity-cultured shrimp still needs attention. This study’s results can give reference to the toxicological evaluation of ammonia stress in the seawater- and low salinity-cultured shrimp.

## Data Availability

Data will be made available upon request.

## Supplementary Materials

The following supporting information can be downloaded at: https://www.mdpi.com/article/10.3390/biology13040281/s1, Table S1: Primer sequences used in this study.
