# Supplementary material for: Toxicity of Ammonia Stress on the Physiological Homeostasis in the Gills of Litopenaeus vannamei under Seawater and Low-Salinity Conditions
Source: Biology (Basel). 2024 Apr 21;13(4):281. doi: 10.3390/biology13040281 (PMC11048301; doi:10.3390/biology13040281)
Supplement: Supplementary file 1 [file biology-13-00281-s001.zip › biology-2953979-supplementary.pdf]

Supplementary materials

Table S1 Primer sequences used in this study.

Gene name	Forward primer (5'-3')	Reverse primer (5'-3')
nrf2	GATGAGAAGCGAGCCAGAGCG	GCCGTCGGATGTCTCGGATAA
sod	GACACGACCATTAGCCTGTACGAC	CAGCGTTGCCAGTAGCGAGTG
cat	TGATCGCTACAACAGTGCTGATGAG	ATGTTCTCCACCAAACGCTGACG
gpx	TCTGAGCGGCGAGATGGTGTC	CTGGTGGAAGTCCCTGGTGGTC
hsp70	TGTGCCTGCCTACTTCAACGATTC	CGCTCACCGCCAACCTTCTTG
hsp90	TTCGGCGTGGGCTTCTACTCC	TACTGCTCGTCGTCGTTGTTCTTG
trx	TCCGTCCTCCTCTTCCCACATTC	GGCGTAGAAGTCGATGACAACCAG
bip	CCAGCACGACATCCAGTTCTTCC	CCTCCGCAGCAAACACCTTCTC
ire1	GGTACATTAGGTTCTCGTCCGTCAC	AATTCCTCTGGTGTTGCCTTAGCC
xbp1	CGCCTGCTGAGGATGACCTTATTAC	GCCTACTGGTGATGTGTCCTTAACG
casp-9	ATGGCTCGTGGTTCATTCAG	CATCAGGGTTGAGACAATACAGG
casp-3	AGACGGACAGCATACAGGAGGAC	CTCGGCCAAGAAGTGGATGAAGAC
jnk	AAAGGGCATACAGGGAGTTCAAGC	GGCACAGGTTGGCATCCATCAG
cytp450	CAACGACACCATCAGGAGCAGAC	CAGCAGCAGGTCCAAGAAGGC
gst	ACGAACACTACGAACAGAAGGATGC	GCCAGGAAGTCGATGTAGGTTAGC
ugt	CTGCTTCTGCTGATCCTGCTGAC	CCTCTCCTACACCCTCTTTCTCCTC
sult	CTGCACCACATGAGTCGTCTCTTC	GTGTAGCCAGTACGGTCCTTGAAC
pdh	TCAGCCTCAACCAACTACTACACTC	GCCTCCTTCACACTCAGTACATCC
hk	ACCTGCTGCTGGTTCACGATG	GCTGCTGCCTCCTCCAAGTG
pk	GCCAGACAGTGCCATCTCTACC	TGCCAGCCAGTCACCACAAC
ldh	GATCGGCTCAGGCACCAACC	GCAACATTAACACCAGACCAGACAG
mdh	CTCTTCCACCCAGTTCCCAGATG	GACAACTTACGAGCGGCAATGAC
cs	GCTCGGTTCCATCCATCCTCTG	ACGCCTTCTGTTGGTGTCCTAAG
sdh	TTCCTGGCACTCACTATGACTGTTC	GATGAAGTAGCAGAGACCTCCCAAG
idh	ACGGAGACCAATACAAGGCTACTG	TGAGTGTGCGAAGGAACGGATAG
odh	GCAAGGCATAATCAGGGCATATCAG	CATAGGAGCGGACTACTGTTCTGG
ndh	CTTCTTGGTTCGGTGCTTGAATGG	AGCAGCCTCTGAAGAGTATTGGTTG
ATPase	CACCATCATCAACCAGAAGCGATTC	GGAGCAGCATCAGAGGCAGTG
cco	ATGCCAGGTGTCCGCTTCAAG	AAGGGTCAACTTGTTCCAGTCTCC
coi	CCCAGATATAGCCTTCCCTCGAATG	GCGTGAGCAATACTGGCAGATAAAG
cytc	TCGACGTGTACCTGACCAACCC	TTCGCCTGGCTTCCTCTTCCTC
nka-a	TGAAATCGTGTTTGCCCGTACCTC	ACCATCACCAGTTACAGCCACAATG
nka-β	GAACCCAGCCGACGAAGAATACG	CAGCAACAATAGGTGGCAGGTAGC
ca	CCTATTCTGGCTCCCTCACTACCC	GTTCATCCTCTGGGCAACACTCG
aqp	GCAGCCATCTTGAAGGGAGTGAC	ACGAGGACGAAGGTGATGAGGAG
clc	GCCGATAGGAGGTGTGTTGTTCAG	CGAAGAAGCCACGCCAGTAGTTC
ccp	CCCTGAGACCTCTGTTTGCTGTG	TGGCAGTGTTCTTCGCATGTTCC
β-actin	TCGCTCCCTCCACCATGAAGATC	CTCCTGCTTGCTGATCCACATCTG
